# EEG Analysis of the Brain Activity during the Observation of Commercial, Political, or Public Service Announcements

**DOI:** 10.1155/2010/985867

**Published:** 2009-12-24

**Authors:** Giovanni Vecchiato, Laura Astolfi, Alessandro Tabarrini, Serenella Salinari, Donatella Mattia, Febo Cincotti, Luigi Bianchi, Domenica Sorrentino, Fabio Aloise, Ramon Soranzo, Fabio Babiloni

**Affiliations:** ^1^Department of Physiology and Pharmacology, University of Rome “Sapienza”, P.le A. Moro 5, 00185 Rome, Italy; ^2^IRCCS Fondazione Santa Lucia, Laboratory of Neuroelectrical Imaging, Via Ardeatina 354, 00179 Rome, Italy; ^3^Department of Computer Science and Informatics, University of Rome “Sapienza”, Via Ariosto 25, 00100 Rome, Italy

## Abstract

The use of modern brain imaging techniques could be useful to understand what brain areas are involved in the observation of video clips related to commercial advertising, as well as for the support of political campaigns, and also the areas of Public Service Announcements (PSAs). In this paper we describe the capability of tracking brain activity during the observation of commercials, political spots, and PSAs with advanced high-resolution EEG statistical techniques in time and frequency domains in a group of normal subjects. We analyzed the statistically significant cortical spectral power activity in different frequency bands during the observation of a commercial video clip related to the use of a beer in a group of 13 normal subjects. In addition, a TV speech of the Prime Minister of Italy was analyzed in two groups of swing and “supporter” voters. Results suggested that the cortical activity during the observation of commercial spots could vary consistently across the spot. This fact suggest the possibility to remove the parts of the spot that are not particularly attractive by using those cerebral indexes. The cortical activity during the observation of the political speech indicated a major cortical activity in the supporters group when compared to the swing voters. In this case, it is possible to conclude that the communication proposed has failed to raise attention or interest on swing voters. In conclusions, high-resolution EEG statistical techniques have been proved to able to generate useful insights about the particular fruition of TV messages, related to both commercial as well as political fields.

## 1. Introduction

Every day we are exposed to several solicitations for purchasing products, voting or supporting particular politicians and even improving our life style. Such pressure has become usual, being mediated by all the current media available, video, audio, and even internet. How and to what extent these messages could be detected and recognized by our brain is still not well understood. In fact, the study of brain responses to commercial and political announcements has been measured mainly by the hemodynamic responses of the different brain areas, by using the functional Magnetic Resonance Imaging devices (fMRI). However, both the stimuli and the relative brain responses have rapidly shifting characteristics that are not tracked by the evolution of the hemodynamic blood flow, which usually lasts 4–6 seconds. Different brain imaging tools, mainly EEG and Magnetoencephalography, exhibit a sufficient time resolution to follow the brain activity at an expense of a coarse level of spatial resolution with respect to the fMRI. In fact, during those last ten years, the use of the high resolution EEG techniques has retrieved an increased amount of information related to the brain during activities related to complex cognitive tasks, such as memory, visual attention, short-term memory, and so forth [1–3]. 

Starting from the interesting characteristic of the high resolution EEG techniques for the tracking of brain activity, the present work would like to describe neuroelectric-based methodology for the assessment of the efficacy of commercial, politic, and Public Service Announcements (PSAs). 

The aim of the brain imaging techniques applied to the fruition of commercial advertizing is to understand mechanisms underlying customer's engagement with brand or company advertized [4–6]. In particular, the issue is to explain how the exposure of subsequent film segments is able to trigger in the consumer mind persisting stimuli leading to interest, preference, purchase, and repurchase of a given product. In the last decades, several authors have investigated the capability of subjects to memorize and retrieve sensible “commercial” information observed during a TV spot [7–12]. 

Recently, a growing number of research laboratories are involved in recognizing the cerebral areas activated during the observation of figures and videos showing politicians, a field that the most people call Neuropolicy. This intense scientific movement has encouraged some companies and universities to seriously take an interest about the cerebral activity during fruition of politicians' images and TV commercials [13, 14]. 

Local governments of European countries, and also across the world, are called to disseminate information about health risky habits promoting instead healthy life style in order to improve the health of their citizens. In this context PSAs are noncommercial broadcast advertizemnets intended to modify public behavior. PSAs are at the core of many public health campaigns against smoking, fatty foods, abuse of alcohol, and other possible threats for the health of citizens. But the content of these PSAs could be also directed for the promotion of “positive” social collective behavior, for instance, calling against racism, supporting the integration of different cultures in the country, or promoting a healthy drive style, for the road security. Therefor effective PSAs provide a great public health benefit [15, 16]. However, the lack of reliable, quantitative, and objective means of advertizement evaluation is one of the impediments to better PSA outcomes. In addition, not well-designed PSAs are going to have counter effects with respect to their desired goals [17]. 

The purpose of this paper is to illustrate the potential of the High Resolution EEG techniques when applied to the analysis of brain activity related to the observation of TV commercials, political advertizing, and PSAs to localize cerebral areas mostly emotionally involved. In particular, we would like to describe how, by using appropriate statistical analysis, it is possible to recover significant information about cortical areas engaged by particular scenes inserted within the video clip analyzed. The brain activity was evaluated in both time and frequency domains by solving the associate inverse problem of EEG with the use of realistic head models. Successively, the data analyzed were statistically treated by comparing their actual values to the average values estimated during the observation of the documentary. Statistical estimators were then evaluated and employed in order to generate representations of the cortical areas elicited by the particular video considered.

## 2. Materials and Methods

The whole dataset is composed by EEG registrations of 13 healthy subjects (mean age 30 ±4 years) watching a documentary of 30 minutes intermingled by a TV commercial [18], and 10 subjects were involved in the observation of a documentary of 15 minutes intermingled with a couple of video clips supporting the Italian Prime Minister (politic announcement) and a campaigns against smoking (PSA). Each subject is exposed to the observation of a same documentary. Subjects were informed about the fact that the EEG recordings will be related to the observation of the brain activity during the documentary and TV commercial, political, and PSAs videos. The entire procedure was authorized by the local ethical committee at the recording site, and informed written consensuses to the recording procedures were taken before the EEG recordings. Subjects were instructed to pay attention to the material showed on the screen during the entire projection. The videos related to products, political support, and PSAs were inserted at the middle of the documentary. 

In order to enhance the poor spatial content of the EEG activity, we employed the High Resolution EEG technologies [19, 20] to detect cortical areas involved in the task. Basically, these techniques involve the use of a large number (64–256) of scalp electrodes and rely on realistic MRI-constructed head models [21, 22] and spatial deconvolution estimations, which are usually computed by solving a linear-inverse problem based on Boundary-Element Mathematics [23, 24]. Subjects were comfortably seated on a reclining chair, in an electrically shielded, dimly lit room. A 64-channel EEG system (BrainAmp, Brainproducts GmbH, Germany) was used to record electrical potentials by means of an electrode cap, accordingly to an extension of the 10–20 international system. In the present work, the cortical activity was estimated from scalp EEG recordings by using realistic head models whose cortical surfaces consisted of about 5000 triangles uniformly disposed. The current density estimation of each one of the equivalent electrical dipole of the underlying neuronal population was computed by solving the linear-inverse problem according to the techniques described previously in this papers [18, 25, 26]. Thus, a time-varying waveform relative to the estimated current density activity at each single triangle of the modeled cortical surface was obtained. Such waveform was then subjected to the time-varying spectral analysis by computing the spectral power in the different frequency bands usually employed in EEG analysis, that is, theta (4–7 Hz), alpha (8–12 Hz), beta (13–24 Hz), and gamma (24–45 Hz).

However, the estimation of the spectral power do not convey information about the significance or nonsignificance of what have been computed. This significance has to be brought to the analysis by taking into account the *z*-score transformation of the cortical power spectra source imaging obtained. In fact, if a cortical area shows an increased activity during the period of the video to be tested, this increment has to be contrasted with the average power spectra activity observed during the documentary period. The *z*-score variable is then obtained by computing the differences between the estimated values (power spectra in the commercial advertizing, political announcement, or PSA) in the video and the average power spectra activity during the documentary. This difference is successively divided by the standard deviation of the power spectra estimated during the documentary period. 

Together with the statistically significant cortical source imaging applied in all the investigated videos, we would like to track specifically the changes in the power spectral intensity of the EEG channels during the videos observation. In order to do that, first we selected the set of the frontal leads, including Fp1, Fp2, F1, F3, Fz, AFz, F2, F4, AF1, and AF2. Then, we filtered the observed EEG activity in two main frequency bands: theta (4–7 Hz) and beta and gamma bands (13–40 Hz) [27]. This filtering procedure was justified by the fact that the theta frequency band is mainly involved in the memorization processes, while the beta and gamma frequency bands are instead advocated for the attention engagement. To summarize the activity from all these electrodes, the Global Field Power (GFP) was then computed. This is a measurement introduced by Lehmann and Michel some decades ago to summarize the overall activity over the scalp surface. GFP is computed from the entire set of electrodes by performing the sum of the squared values of the EEG potential at each electrodes, resulting in a time-varying waveforms related to the increase or decrease of the global power in the analyzed EEG. Since the data were estimated from two EEG datasets, one filtered 4–7 Hz and the other filtered between 13–40 Hz, we obtained two time-varying waveforms: the GFP filtered in the 4–7 Hz and the GFP filtered in the 13–40 Hz. Successively, on these waveforms we apply the *z*-score transformation by estimating the average and the standard deviation of the GFP values during the documentary. Values of *z*-score transformation higher than 2 suggest statistical significance differences between the value of the variable estimated and the baseline considered, at the 5% level. 

In order to present these results relative to the entire population, we needed a common cortical representation to map the different activated areas of each subject. For this purpose we used the average brain model available from the McGill University website to display the cortical areas that are statistically significantly activated during different experimental conditions in all subjects analyzed. In this case, we are able to average the single subject result of the *z*-score test. In fact, we highlighted in yellow a voxel of the average brain model if it was a cortical site in which a statistical significant variation of the spectral power between the experimental conditions was found in all the subjects; if such brain voxel was statistically significant in all but one of the subjects analyzed, we depicted it in red. In all the other cases, the voxel was represented with a gray color. Only the statistical significant variation of such spectral power when compared to the documentary period was highlighted in color. Statistical significance threshold was set at *p* < 0.05, which Bonferroni corrected for multiple comparisons.

### 2.1. Commercial Advertizing

After the EEG registration related to the commercial advertizements each subject was recalled in laboratory where an interview was performed asking if he/she usually drinks beer or light alcohol at least once per week. If yes, subjects were considered within the dataset of “drinkers” in opposition to the dataset of “no drinkers”. In order to increase the sensitivity of the analysis performed, only the EEG spectral analysis for the “drinkers” was analyzed and presented here.

## 3. Results

**Figure 1:**
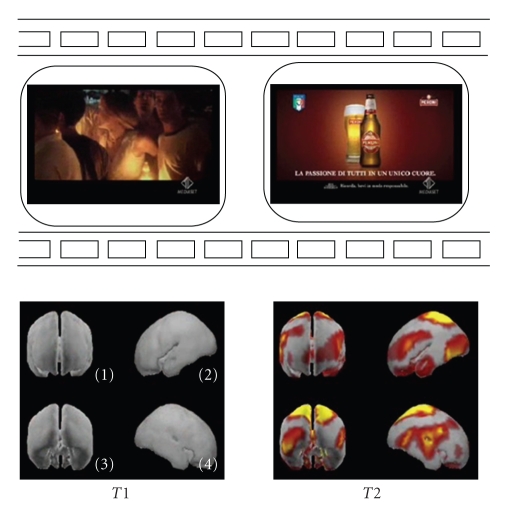
The track of the mean cortical activity of the group of “drinkers” in the theta frequency band spot. The statistical significant activity in this population is shown in 2 panels each representing subsequent film segments of a TV spot with corresponding brain activity. Temporal axes beat the spot in correspondence to the beginning (*T*1) and the end (*T*2) of the entire film sequence: time in seconds. Note in the left lower panel the different brain views are relative to the brain seen from the back (1), left (2), front (3), and right (4).

Of the 13 subjects recorded, only seven are “drinkers”. Hence, the successive analysis and results are presented for seven of such subjects. We summarized all results for the “drinkers” group in a couple of figures showing the statistically significant differences of cortical activation concerning this dataset in the theta frequency band (4–7 Hz), being the data regarding the alpha frequency band equivalent to the theta band. Figure 1 presents the *z*-score average activity of the drinkers population during the observation of a couple of frames of the commercial advertizing proposed, at time: *T*1 = 10 seconds and *T*2 = 28 seconds (the entire duration of the spot is 30 seconds). Note in the left lower panel the different brain views are relative to the brain seen from the back (1), left (2), front (3), and right (4)*. *It is possible to note that the brain is presented from different perspectives in the lower panels, while in the upper panels there are the video frames observed by the subjects. The gray color represents the absence of the statistical differences between the cortical activity in the analyzed frequency band (theta in this case) during the video clips observation and the documentary. The color zones instead presented the cortical areas in which such spectral activity differs in the population analyzed. 

By examining this strip, it results evident how the temporal evolution of the mean cortical activity changes according to the images viewed by the subjects. In particular, an enhancement of cerebral activity is suggested by the result of the application of the statistic tests at the beginning (*T*1) and at the end of the commercial presented (*T*2). The final part of the commercial spot attracts the interest in all the population analyzed, as the large cortical areas depicted in yellow and red suggested. It could be hypothesized that the initial part of the TV spot presented fails to attract the attention of the experimental group, as the brain activity result is similar to that generated during the observation of the documentary. This could be used in future to better tailor the TV spots by removing the parts of them that were unable to attract the attention of the audience. 

**Figure 2:**
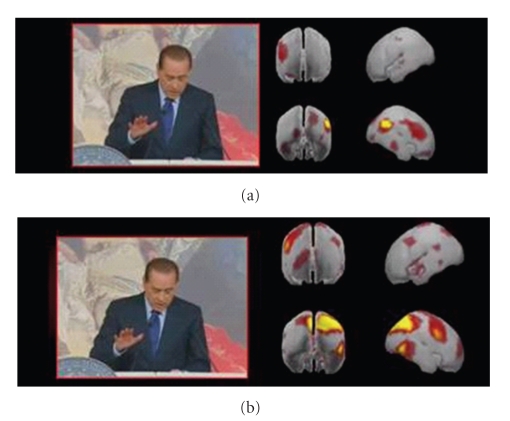
Analysis of a speech by the Italian Prime Minister: panel (a) presents the cerebral activity related to the population of swing voters in the theta frequency bands. Note the four brains depicted at the right of each frame are relative to the four different views of the brain surface from different points of view; same convention of Figure 1, panel (b) shows the cortical activity in the same frequency band for the supporters of the Prime Minister. Cortical areas depicted in red and yellow highlight those zones in which there is an enhancement of cerebral activity, when compared to a resting state.

The applications of the abovementioned technology to the evaluation of a TV speech of the Italian Prime Minister are shown in Figure 2. 

Such figure shows the cerebral activity observed in two groups of people divided as swing voters (panel a) and the “supporters” of the Italian Prime Minister (panel b). The figure presents the statistically significant activity in the analyzed groups in the theta frequency band for a particular time frame of the speech. The brain activity is presented as seen from different perspectives (left, right, front, and back). The brain activity observed for the supporters (panel b) was characterized during all the speech by power spectral activity significantly larger than those obtained in the documentary. On the contrary, swing voters are relatively less attracted by the speech, since they had the brain activity not different from that of the documentary for the major part of the speech. 

Hence, the results would suggest an overall efficacy of the communication generated by the video for the second group of subjects (panel b) with respect to the “perceived” communication offered by the prime minister's speech by the first one (panel a). A possible interpretation of these results is that analyzed speech could only intensify the supporters' idea leaving the swing voters neutral. 

**Figure 3:**
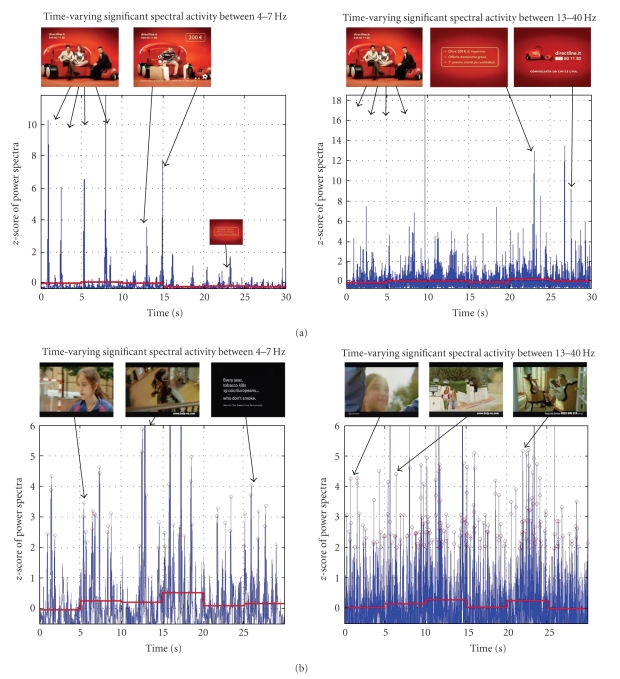
Representation of the filtered GFP related to frontal electrodes in the theta band (left panel) and beta and gamma bands (right panel) for the analyzed population during the observation of a commercial (row a) and a Public Service Announcement (row b) against the smoking. On the *x*-axis, there is the time duration of the spot; on the *y*-axis there is the *z*-score value of the GFP considered. Values greater than 2 mean a difference in power spectral activity during the PSA when compared to those of the documentary that are statistically significant at 5%. The red line is the average *z*-score values of the GFP in blocks of five seconds of spot.

Another example of the application of the neuroelectric brain imaging technology applied to the case of the commercial and PSAs is represented in Figure 3. In such figure the time-varying changes of the filtered GFP in the theta (4–7 Hz) and in the beta and gamma bands (13–40 Hz) during the observation of a 30-second spot related to a commercial (row a) and a PSA against smoking (row b) are presented. It is interesting to note how in both cases the spectral activity of the frontal leads showed values of *z*-score over 2 for many instants. In such a case it could be suggested that the spectral activity in those time frames exceeds statistically the spectral activity observed during the vision of the documentary. 

**Figure 4:**
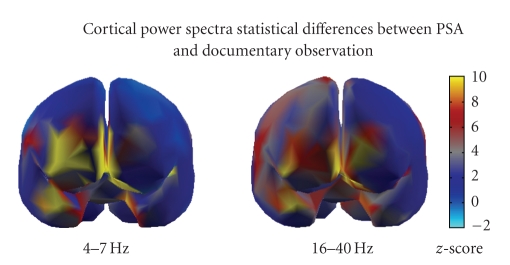
Brain activity related to the observation of the PSA when compared to that of the documentary. The brain is viewed from a frontal view. The color map is relative to the *z*-score values of the power spectra EEG activity during the PSA when compared to the documentary. *Z*-score values different from the blue means significant statistically activity. It is possible to note that in the theta frequency band, the right prefrontal cortex is activated while in the beta and gamma frequency bands the activation is rather symmetrical.

The brain activity observed during this PSA spot is instead presented in Figure 4, in which two cortical maps of the differences between the spectral activities during the spot when compared to those of the documentary are represented. Figure 4 shows the *z*-score maps during the PSA, where it is possible to note a precise activation of the frontal lobes during the 30 seconds of the spot. Also in this case it is interesting to note that the brain activity during the PSA is relevant in the prefrontal areas, and it is symmetrical in the beta and gamma frequency bands while located in the right frontal hemisphere for the theta band.

## 4. Discussions

Thanks to the high resolution EEG techniques, we tracked subjects' brain activity during visualization of a commercial: in such manner, it has been possible to obtain a global measure of the reconstructed cortical signals by means of a simple graphic tool which allows us to distinguish the activity of different cortical areas. The abovementioned results allow us to comment on temporal and spatial events observed. In fact, it is worth noticing that the principal areas of statistical differences in power spectra in the “drinkers” condition are located almost bilaterally in the prefrontal BAs 8 and 9 as well as in the parietal BA 7. As presented in previous works performed both with EEG analysis [18] and MEG recordings [8], the observed phenomena suggest an active role of the prefrontal and parietal areas in coding of the information that will be retained by users from the TV commercials. In particular, activations of these cortical areas can be associated with attentional and memorization processes. The present work intends to stress the useful properties of the high resolution EEG technologies: this tool is able to help us in observing and analyzing the temporal trend of the cortical activities thanks to a high-temporal and spatial resolution allowing us to distinguish changes of activation of ROIs corresponding to different cortical areas. The reconstruction of the cortical activity by means of the high resolution EEG technique and by combining the above statistic treatment of our data allowed us to track subjects' brain activity during visualization of the commercial. In such a way for each film segment of the clip it was possible to distinguish cortical areas that were differently activated when compared to those of the observation of the documentary. This could be useful in the evaluation of the cortical responses to particular types of visual solicitations, performed by film, commercial clips, or faces of politicians, which at the moment is a field largely unexplored by the neuroscience. In fact, the big attention paid to political scenes originates from the experimental result that decisions based on “superficial” observation could predict the elections' results, linearly correlated with the candidate's margin of victory with a precision of 68.8% [28]. The consequence of this observation is that the recognition and liking of the politician's face is a principal factor for the choice of the citizen more than “rational” considerations. Such phenomenon has been also confirmed by subsequent studies published in international scientific literature [29] and newspapers [30] suggesting that the scenic presence by itself mostly influences the decision of voting besides the fact that men and women can elicit a different kind of engagement according to the figure they saw. These results are surprising if we think about the USA midterm elections of 2006 when candidates and their supporting groups spent about 1 billion dollars in advertizements in order to inform electors about their political affiliations, qualities, and ideas. After predicting the electoral results [28, 29], the subsequent pass of this research has been to understand whether in this immediate decision the positive effects were prevailing (i.e., face pleasantness as well as his/her adaptation to particular a priori requirements demanded by the candidate) when compared to the negative ones. This was then studied in literature [29] and its result was that the effect of an emotionally “negative” judgement towards a candidate is an prevailing reason in his/her defeat (even in a contest of simulated elections) with respect to the fact of simply being “less attractive” then the other candidate. These data could also suggest that the cerebral activity, generated from an emotional state of “rejection” of the candidate, is completely different from the one generated from an emotional state of acceptation or satisfaction of the same one. Another interesting aspect is that results obtained by means of analyses performed on behavioral data reveal how decision makers sometimes have already made up their minds at an unconscious level, even when they consciously indicate that they have not yet decided. From the traditional political research, that is, performed without using cerebral measurements techniques, it was already known that the negative vote plays also an important role in the final vote decision. In this contest, having a measure of the emotional state of people observing a candidate's face assumes always more importance.

## 5. Conclusions

We employed advanced techniques for the tracking of brain activity during the observation of videos of different emotional natures: commercial, PSAs, and even political speech. The techniques here employed encompass the use of descriptors of the frequency contents of the EEG signals up to the statistical mapping of the cortical activity, during the observation of the proposed clips [31–36]. In all the cases presented here, it is possible to localize rather precisely the cortical areas involved in the processing of the particular video material proposed. The application of these techniques to the PSAs and to the political speech is still in its infancy, but in a near future could bring a neuroscience perspective in an already mature communication field.
